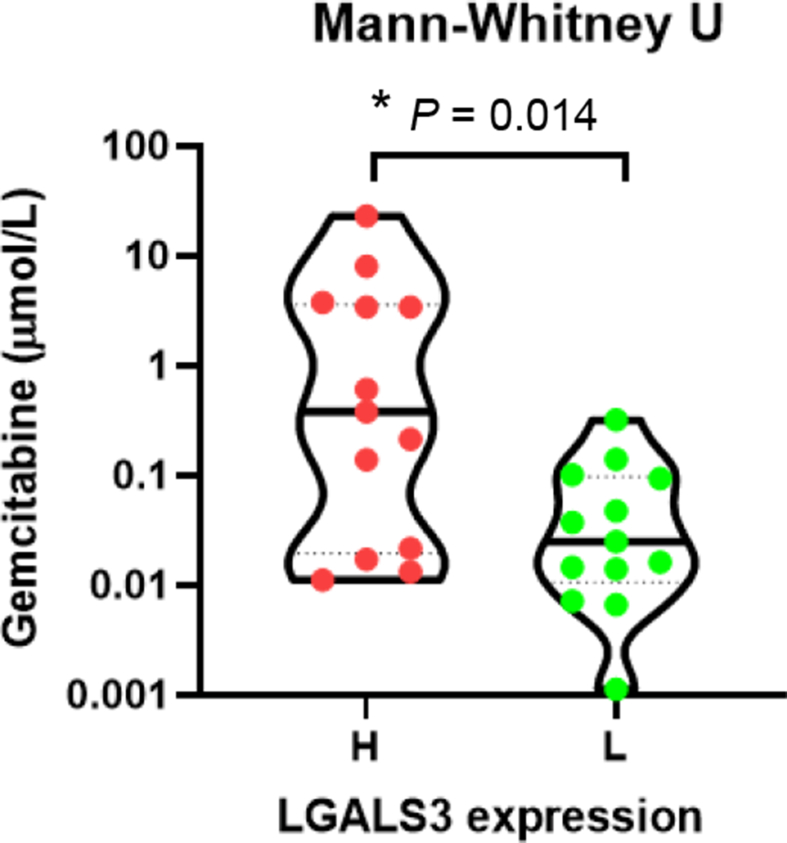# Galectin-3 in tumor-stromal cells enhances gemcitabine resistance in pancreatic adenocarcinoma by suppressing oxidative phosphorylation

**DOI:** 10.1016/j.gendis.2025.101702

**Published:** 2025-05-29

**Authors:** Yaheng Wu, Guo An, Jia Tong, Wenlong Zhang, Zhihua Tian, Bin Dong, Xijuan Liu, Lin Zhao, Chunxiang Ye, Jingtao Liu, Wei Zhao, Huachong Ma

**Affiliations:** a Beijing Luhe Hospital, Capital Medical University, Beijing 101149, China; b Department of Acute Abdominal Surgery, Beijing Chaoyang Hospital, Capital Medical University, Beijing 100020, China; c Key Laboratory of Carcinogenesis and Translational Research, Department of Laboratory Animals, Peking University Cancer Hospital & Institute, Beijing 100142, China; d Department of Geriatric Medicine, Shandong Provincial Hospital Affiliated to Shandong First Medical University, Shandong First Medical University, Jinan, Shandong 250021, China; e Key Laboratory of Carcinogenesis and Translational Research, Department of Central Laboratory, Peking University Cancer Hospital & Institute, Beijing 100142, China; f Key Laboratory of Carcinogenesis and Translational Research, Department of Thoracic Surgery II, Peking University Cancer Hospital & Institute, Beijing 100142, China; g Department of General Surgery, Beijing Chaoyang Hospital, Capital Medical University, Beijing 100020, China; h Key Laboratory of Carcinogenesis and Translational Research, Department of Pharmacology, Peking University Cancer Hospital & Institute, Beijing 100142, China; i Key Laboratory of Carcinogenesis and Translational Research, Department of Clinical Laboratory, Peking University Cancer Hospital & Institute, Beijing 100142, China; j Cardiff China Medical Research Collaborative, Division of Cancer and Genetics, Cardiff University School of Medicine, Cardiff CF14 4XN, UK

**Keywords:** CCL2, Galectin-3, Gemcitabine, Pancreaticstellate cells, Pancreatic adenocarcinoma

## Abstract

Galectin-3 (Gal-3) plays a multifaceted role in the development and progression of pancreatic adenocarcinoma (PAAD), which is associated with a poor prognosis. Its interaction with tumor microenvironment cells has been reported. However, the Gal-3-mediated tumor–stromal interaction and induced energy metabolism associated with drug resistance remain unknown. Our previous study has reported that Gal-3 secretion from tumor cells and inflammatory cytokine dependency are therapeutic targets. In this study, we revealed that the expression of Gal-3 was not only remarkably up-regulated in tumors but also significantly associated with the tumor-associated fibroblasts of PAAD patients. A coculture model of PAAD cells and pancreatic stellate cells revealed that Gal-3 mediated the Ca^2+^/−calcineurin–NFAT pathway to increase the transcription of CCL2 and BSG in tumor-associated fibroblasts. These findings ultimately lead to the observation of low energy metabolism in tumor cells. Particularly, mitochondrial oxidative phosphorylation was functionally arrested in Gal-3-high tumor cells, as demonstrated by a lower oxygen consumption rate and mitochondrial ATP production through abnormal mitochondrial morphology. The inhibition of the CCL2-CCR2 and PPIA-BSG pathways indicated the restoration of gemcitabine sensitivity when drug resistance was elicited by Gal-3. Oral administration of the natural Gal-3 inhibitor modified citrus pectin extract (MCP) showed therapeutic effect for Gal-3-activated tumors and stromal cells in orthotopic pancreatic xenograft models. Hence, our findings offer insights into the fact that low mitochondrial metabolism is dependent on Gal-3 activation-mediated gemcitabine resistance through tumor–stromal interactions.

## Introduction

Pancreatic cancer accounts for nearly 3% of all cancer cases and represents a relatively common malignant tumor with increasing incidence and mortality rates.^1^ This type of malignant tumor is often diagnosed at an advanced stage and has a poor prognosis, and the 5-year survival rate is only 5%.^2^ According to statistics, there were 50,000 deaths in the US and 106,300 deaths in China due to pancreatic cancer in 2023.^2^^,^^3^ Over the past 25 years, the incidence of pancreatic cancer has continued to rise, which has sparked widespread concern about this public health issue.

Although imaging studies (CT, MRI, *etc*.) and pathological analysis have become essential diagnostic tools for pancreatic cancer,^4^ recent research has increasingly recognized the potential of galectin-3 (Gal-3) as an effective early diagnostic molecular target.^5^ Gal-3 is a chimeric protein located on the 14th chromosome. It has been proven to participate in various biological processes, including cell proliferation, apoptosis, and immune responses.^6^^,^^7^ Compared with those in healthy individuals and patients with benign pancreatic disease, Gal-3 expression levels are significantly higher in pancreatic cancer tissues and patient sera, providing evidence for its use as a potential biomarker.^8^ Gal-3 is a multifunctional driver of pancreatic cancer progression. Overcoming the stromal and immunosuppressive barriers in pancreatic cancer remains critical for translating galectin-3-directed strategies into clinical benefits. Gal-3 interacts with the dense stromal environment characteristic of pancreatic cancer. It modulates extracellular matrix remodeling, activates cancer-associated fibroblasts, and promotes angiogenesis, fostering a pro-tumorigenic niche.^9^ Gal-3 enhances metastasis through glycan-mediated adhesion in the vasculature and further facilitates epithelial–mesenchymal transition and cell migration by interacting with integrins and matrix metalloproteinases.^10^ For therapeutic targeting investigation, GM-CT-01 (a galactomannan) and modified citrus pectin (MCP) block galectin-3 carbohydrate-binding domains, showing preclinical efficacy in reducing tumor growth and metastasis.^11^^,^^12^ Therefore, Gal-3 represents a promising therapeutic target for intervening in the progression of pancreatic cancer.

Our previous studies have demonstrated that Gal-3 could increase the malignancy of tumor cells and activate collagen and fibrin crosslinking in the stroma. In addition, Gal-3 promotes the release of many inflammatory cytokines, such as interleukin (IL)-6 and IL-8, by stromal cells,^12^ which accelerates tumor progression through deterioration of the tumor microenvironment.^13^ Additionally, we found that MCP, a natural inhibitor of Gal-3,^14^ inhibited tumor growth in a mouse xenograft model of pancreatic cancer. This study provides new ideas for tumor treatment via dietary interventions. However, there are few reports on the role of Gal-3 in chemotherapy resistance in pancreatic cancer, so an in-depth exploration of this topic is urgently needed.

This study aims to further elucidate whether the inhibition of Gal-3 expression in tumor cells or stromal cells can be combined with gemcitabine to prevent pancreatic cancer progression effectively. We evaluated Gal-3 levels in various types of PAAD samples and subsequently established stably transfected pancreatic cancer cell lines. Based on a previous report, gemcitabine sensitivity in different cell lines was determined to assess the relationship between Gal-3 expression and drug resistance.^15^^,^^16^ These results indicate that the inhibition of Gal-3 expression may reduce the dose dependence of pancreatic tumors on gemcitabine chemotherapy, thereby increasing treatment efficacy. Furthermore, we explored the effects of Gal-3 on mitochondrial oxidative phosphorylation and reactive oxygen species (ROS) levels during the substrate end of the respiratory chain in the mitochondrial membrane in PAAD cells and the associated pathways in gemcitabine chemotherapy. Fibroblast-induced drug-resistant tumors are significantly reduced through the C–C motif chemokine 2 (CCL2)-C-C motif chemokine receptor 2 (CCR2) axis. This reduction occurs in a multifactorial manner and involves the calcium-dependent peptidylprolyl isomerase A (PPIA)-basigin (BSG, also called CD147) pathway and the dephosphorylation of nuclear factor of activated T-cells 1 (NFAT1) via intracellular mechanisms. The consequence of such a phenomenon is that it enables these cells to effectively counter the DNA damage caused by gemcitabine. Our research provides significant insights for future dietary interventions and the development of novel agents, opening new avenues for improving patient prognosis.

## Materials and methods

### Establishment and culture of Gal-3 stable cell lines

Human pancreatic cancer cell lines (BxPC-3 and Panc-1) and human pancreatic cancer stroma-associated immortalized fibroblasts, called pancreatic stellate cells (HPSCs), were established and obtained from The University of Texas MD Anderson Cancer Center.^17^ All the cell lines were authenticated via short tandem repeat (STR) DNA fingerprinting performed by the Cell Line Core. The cells were maintained in either RPMI-1640 or Dulbecco's modified Eagle's medium supplemented with 10% fetal bovine serum, 100 U/mL penicillin, and 100 mg/mL streptomycin (Thermo Fisher Scientific, Waltham, Massachusetts, USA) as suggested by the vendor. Concerning lentiviral systems, stable cell line establishment was performed via overexpression constructs, including Gal-3-overexpressing cell lines (Panc-1-OE, BxPC-3-OE, and HPSC-OE), along with their respective control cell lines (Panc-1-vector, BxPC-3-vector, and HPSC-vector). The efficiency of gene targeting in these cell lines has been shown in previous reports.^12^

### Sphere formation assay

The cells were counted and plated at low density (approximately 100 cells per well) in ultralow attachment 96-well plates (Corning Incorporated Life Sciences, Acton, Massachusetts, USA) in serum-free DMEM/F12 medium (a mixture of Dulbecco's modified Eagle's medium and Ham's F-12 nutrient mixture; Invitrogen) supplemented with B27 (1:50; Invitrogen), 20 ng/mL basic fibroblast growth factor, 10 ng/mL hepatocyte growth factor (PeproTech), and 1% methylcellulose (Sigma–Aldrich). HPSCs and specific pancreatic cancer cell lines were added at a 1:1 ratio. The mixture was incubated in a CO_2_ incubator for 2 weeks, and spheres with a diameter ≥100 μm were counted under an Axio Observer A1 inverted microscope (Carl Zeiss Microscopy GmbH, Jena, Germany).^18^

### Flow cytometry analysis

Pancreatic cancer cells were discerned via anti-CD147 antibodies (Cell Signaling Technology, San Diego, California, USA), which were directly labeled with fluorescein via the respective Lightning-Link Conjugation Kits following the vendor's protocol (Innova Biosciences Ltd., Cambridge, UK).^18^ Following incubation at 4 °C in the dark for 30 min, the cells were washed three times with phosphate buffer saline, resuspended in phosphate buffer saline supplemented with 1% fetal bovine serum, and analyzed via flow cytometry (Beckman Coulter, Brea, California).

### Calcium labeling, imaging, and measurement

The cells were rinsed twice with warmed Tyrode's solution (140 mM NaCl, 5.0 mM KCl, 1.0 mM MgCl_2_, 5.5 mM glucose, 10 mM HEPES, and 1.8 mM CaCl_2_; pH 7.2) and labeled with Fluo-4/AM (Invitrogen, USA) in the same solution at room temperature for 15 min. After washing, fluorescence was measured at room temperature via a confocal microscope (LSM 780, Lecia) equipped with a 40 × 1.3 NA oil immersion objective for imaging. Frames of 1024 × 1024 pixels were captured at intervals of 5 s/frame for 5 min. The total number of frames and the duration of imaging were determined via ImageJ software (http://rsb.info.nih.gov/ij/) and used to measure the intensity of fluorescence of Fluo-4/AM. The change in [Ca^2+^]i was expressed as DF = (F − F0)/F0, where F represents the fluorescence intensity and F0 indicates the resting fluorescence.

### Chromatin immunoprecipitation (ChIP)

The chromatin immunoprecipitation assay was performed with an assay kit obtained from Beyotime Biotechnology (Shanghai, China) according to the manufacturer's protocol. Briefly, 5 × 10^6^ cells were collected and immobilized with 1% formaldehyde at 37 °C for 10 min. The DNA of the cell nuclei was isolated and sonicated to approximately 200–300 bp at 18 MHz. Fragmented chromatin was subjected to immunoprecipitation reactions by employing protein A/G agarose beads that had been preprepared to handle anti-NFAT1 (5 μg) (CST, #4389) or control IgG (5 μg) at 4 °C. The beads were subsequently washed in succession with low-salt immune complex wash buffer, high-salt immune complex wash buffer, LiCl immune complex wash buffer, and Tris–EDTA buffer at 4 °C. NaCl (0.2 M) was used to decrosslink the DNA‒protein complexes through heating (65 °C, 4 h), after which the proteins were digested via protein K treatment. The resulting DNA product was subjected to PCR analysis via CCL2 and BSG promoter-specific primer sequences and run on 1.2% agarose gels.

### Animal experiments

To evaluate the effectiveness of Gal-3 and its inhibitor (MCP) on tumor growth, we established a mouse xenograft model for orthotopic inoculation of pancreatic cancer as follows: First, luciferase-labeled Panc-1 cells (Panc-1-Lu.c) were digested with trypsin and resuspended in phosphate buffer saline (10^6^ cells per 40 μL of phosphate buffer saline) with or without the addition of HPSCs at a 1:1 ratio. The cell suspension was then gently implanted into the orthotopic pancreas of NuNu BALB/c mice (4–6 weeks old; sourced from Beijing Vital River Laboratory Animal Technology Co., Ltd.) with 6 mice per group. Tumor formation was monitored weekly via a noninvasive, real-time cooled bioluminescence imaging system (IVIS 200 Xenogen, PerkinElmer, EU) with XenoLight™ D-luciferin substrate (PerkinElmer, EU) and Living Image software (Xenogen). After 2 months, all the mice were euthanized, and the tumors were excised and weighed. The final tumor volume was estimated via the following formula: tumor volume = [(larger diameter) × (smaller diameter)^2^]/2. The animal studies were approved by the Animal Care and Ethics Committee of Peking University Cancer Hospital (Approval Number: EAEC2018-14), conducted in accordance with institutional guidelines and certified by the Animal Care and Ethics Committee of Peking University Cancer Hospital (certificate number: SYXK 2016-0015).

### Statistical analysis

The results of the cell proliferation assay are presented as mean ± standard deviation. Differences between groups were determined via two-tailed student's *t*-test or the *χ*^2^ test with Fisher's exact probability method via GraphPad Prism software version 7.0. For multiple tests, the Bonferroni‒Holm procedure was applied. The results with a *p*-value <0.05 were considered statistically significant.

## Results

### High Gal-3 expression in multiple tumor types is correlated with a poor prognosis

**Figure 1:**
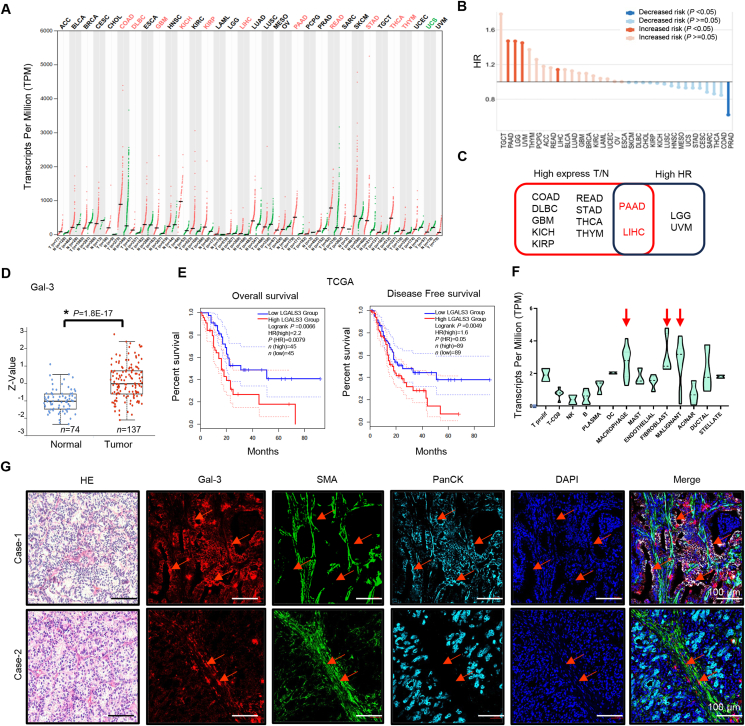
Correlations between galectin-3 expression and the prognosis of patients with tumors and TAFs. **(A)** Analysis of Gal-3 RNA expression levels in multiple tumor types and normal tissues via public RNA sequencing datasets and data from the GEPIA website. The red-marked tumor types presented significantly elevated Gal-3 RNA expression. **(B)** Risk coefficient analysis revealed a significant positive correlation between elevated Gal-3 levels and poor prognosis, with increased clinical risk coefficients in patients with pancreatic cancer, glioma, uveal melanoma, and liver cancer (strong red). A negative correlation is observed in prostate cancer between elevated Gal-3 levels and poor prognosis (strong blue). *p* > 0.05. **(C)** A Venn diagram shows that high Gal-3 RNA expression levels are associated with malignancies in hepatocellular and pancreatic carcinomas. **(D)** Gal-3 expression is significantly elevated in pancreatic cancer at the protein level, as observed in CPTAC protein quantification databases. **(E)** High Gal-3 expression was significantly associated with poor prognosis in terms of both overall survival and disease-free survival. **(F)** Single-cell sequencing analysis revealed that Gal-3 was highly expressed in malignant tumor cells, TAFs, and tumor-associated macrophages in pancreatic cancer (red arrow). **(G)** Multiplex immunofluorescence staining confirmed high Gal-3 expression (red arrow) in tumor epithelial tissue and in fibroblasts within the tumor microenvironment. “∗” indicates statistical significance. Gal-3, galectin-3; TAF, tumor-associated fibroblast.

**Fig. S1:**
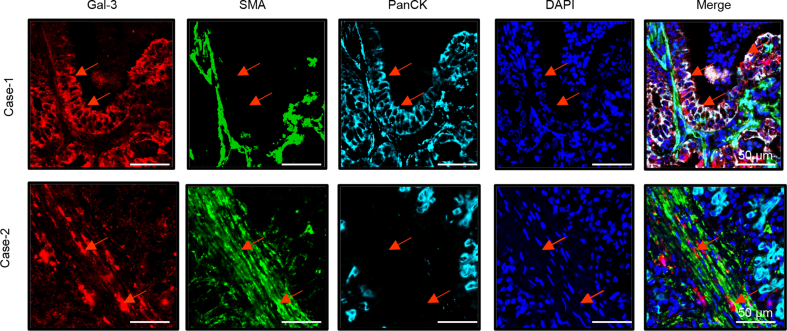


Analysis of Gal-3 expression across multiple RNA sequencing public datasets, combined with data from the GEPIA website, revealed significantly elevated RNA expression levels of Gal-3 in various tumor types, including colon cancer, diffuse large B-cell lymphoma, glioma, kidney cancer, liver cancer, pancreatic cancer, rectal adenocarcinoma, thyroid cancer, gastric cancer, and thymoma, compared with normal tissues (Fig. 1A). Subsequent risk coefficient analysis across multiple tumor types revealed a significant positive correlation between elevated Gal-3 levels and poor prognosis/increased clinical risk coefficients in patients with pancreatic cancer, glioma, uveal melanoma, and liver cancer. Conversely, a negative correlation was observed between the clinical risk coefficient and Gal-3 expression in prostate cancer patients (Fig. 1B). Data from these two databases demonstrated that high levels of Gal-3 RNA were observed in patients with malignancies of the hepatology and pancreatic systems, and there was a notable correlation between these levels and prognosis, with the strongest correlation in patients with pancreatic cancer (Fig. 1C). The RNA sequencing database TCGA (The Cancer Genome Atlas) and the protein quantitation database CPTAC (Clinical Proteomic Tumor Analysis Consortium) revealed that compared with that in normal tissues, Gal-3 expression in pancreatic cancer tissues was significantly elevated at both the RNA and protein levels (Fig. 1A–D). Survival analysis indicated that high Gal-3 expression was significantly associated with poor prognosis in terms of both overall survival and disease-free survival (Fig. 1E). The median survival in the high Gal-3 expression group was 21 months shorter than that in the low Gal-3 expression group. Analysis of multiple single-cell databases for pancreatic cancer revealed that in addition to its expression in malignant tumor cells, high Gal-3 expression was also detected in tumor-associated fibroblasts (TAFs) and tumor-associated macrophages (Fig. 1F). Based on these findings, we conducted immunofluorescence experiments and detected high Gal-3 expression in tumor epithelial tissue (Fig. 1G; Fig. S1, top, case-1), which was also evident in fibroblasts in the tumor microenvironment and was located primarily in the cytoplasm and cell membrane (Fig. 1G; Fig. S1, bottom, case-2). These results indicate that Gal-3 expression is associated with malignancy, poor prognosis, and high levels both in the tumor and tumor-associated stroma of PAAD.

### Gal-3 in tumor cells and TAFs involves multiple pathways

**Figure 2:**
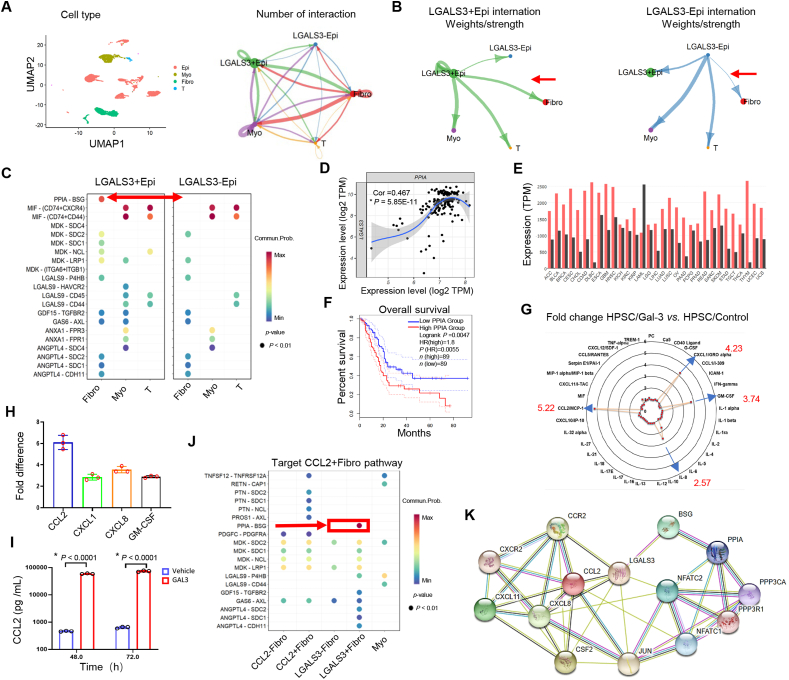
Gal-3 in tumor cells and tumor-associated fibroblasts influences multiple pathways. **(A)** Analysis of the single-cell RNA-sequencing data reveals major cell types in the tumor microenvironment: epithelial cells, myeloid cells, fibroblasts, and T cells. Relationships among each cell cluster, including LGALS3-positive and LGALS3-negative subpopulations. **(B)** Intercellular communication between LGALS3-positive epithelial cells (LGALS3^+^ Epi)/LGALS3-negative epithelial cells (LGALS3^−^ Epi) and neighboring cells. The red arrows indicate significant differences between the Epi and TAF groups. **(C)** Cell–cell crosstalk, which involves ligand–receptor signaling and cytokine secretion/uptake, indicates that the PPIA-BSG pathway is strongly associated with LGALS3^+^ Epi in combination with TAFs. **(D)** Relationships between PPIA and LGALS3 in PAAD from the TCGA database. **(E)** PPIA expression is greater in most solid tumor types than in normal tissues. The red bars represent tumors, and the black bars represent normal tissues. **(F)** Individuals with low PPIA expression have prolonged overall survival in PAAD. Cutoff: 50%. **(G)** The radar chart displays the chromatin immunoprecipitation results for inflammatory factors. The blue arrows show the fold changes in the cytokines that are highly expressed in HPSCs/Gal-3 cells compared with the control. **(H)** Real-time quantitative PCR was used to validate the up-regulation of CCL2 expression at the mRNA level via rGal-3 treatment. **(I)** ELISA further validated the increase in CCL2 expression at the protein level in Gal-3-producing HPSCs. **(J)** Analysis of the single-cell RNA-sequencing data revealed a strong focus on the PPIA-BSG pathway in CCL2-positive fibroblasts. Red indicates a large difference between LGALS3^+^ Epi and LGALS3^−^ Epi. **(K)** Protein–protein interaction network analysis via the STRING database revealed significant interactions between LGALS3, BSG, PPIA, CCL2, CCR2, PPP3CA, and NFATC2. “∗" indicates statistical significance. Gal-3, galectin-3; TAF, tumor-associated fibroblast; LGALS3, lectin galactoside-binding soluble 3; PPIA, peptidylprolyl isomerase A; BSG, basigin; PAAD, pancreatic adenocarcinoma; HPSCs, human pancreatic stellate cells; CCL2, C–C motif chemokine 2; CCR2, C–C motif chemokine receptor 2; PPP3CA, protein phosphatase 3 catalytic subunit alpha; NFATC2, nuclear factor of activated T cells 2.

To investigate the relationship between tumor cells and stromal cells, a cohort of acquired single-cell RNA-sequencing data was analyzed. Four groups of cell types associated with the tumor microenvironment, namely, epicellular cells, myeloid cells, fibroblasts, and T cells, were separated, and the relationship of each cluster, including the lectin galactoside-binding soluble 3 (LGALS3)-positive and LGALS3-negative subpopulations, was explored (Fig. 2A). The differential number of interactions or interaction strengths across various cell types revealed stronger intercellular communication between LGALS3^+^ epithelial cells and their neighboring cells, particularly through cross-talk with TAFs, than between LGALS3^−^ epithelial cells and their neighboring cells (Fig. 2B). Furthermore, to facilitate the transfer of information within the surrounding microenvironment, cell–cell crosstalk involving ligand‒receptor signaling pathways or the secretion/uptake of cytokines was investigated. The results suggested that LGALS3^+^ epithelial cells most significantly highlights the TAF through the PPIA-BSG pathway, although the signaling pathways between the LGALS3^+^ epithelial cells and Myo subsites were low and involved in LGALS9, MDK, GDF, and GAS signaling (Fig. 2C). The RNA-sequencing data from TCGA revealed that PPIA RNA expression was significantly correlated with LGALS3 RNA expression in PAAD (Fig. 2D). Notably, PPIA expression was dramatically up-regulated in almost every solid tumor type compared with normal tissues, except for acute myeloid leukemia (Fig. 2E). In PAAD, individuals with low PPIA expression demonstrated prolonged survival (Fig. 2F).

Additionally, to investigate the critical role of stromal cells in influencing the development of PAAD, the secretion of 36 cytokines from HPSCs was analyzed under Gal-3 treatment via human cytokine protein arrays. Compared with the control, treatment of HPSCs with 500 ng/mL rGal-3 for 48 h resulted in increased secretion of IL-8 (2.57-fold), granulocyte-macrophage colony-stimulating factor (GM-CSF; 3.74-fold), C-X-C motif chemokine ligand 1 (CXCL1; 4.23-fold), and particularly CCL2 (5.22-fold) (Fig. 2G). Real-time quantitative PCR and ELISA further revealed increased CCL2 mRNA and protein levels in HPSCs infected with the Gal-3 lentivirus (Fig. 2H and I). Along with the acquisition of single-cell RNA-sequencing data, the relationship of each clustering target, CCL2-positive fibroblasts, focused on the PPIA-BSG pathway, which plays a critical role in connecting LGALS3-high tumor cells (Fig. 2J). Moreover, we identified the correlations of the targets of interest, namely, LGALS3, BSG, PPIA, CCL2, CCR2, protein phosphatase 3 catalytic subunit alpha (PPP3CA), and nuclear factor of activated T cells 2 (NFATC2), through protein–protein interaction analyses via the STRING website (https://string-db.org/) (Fig. 2K). Based on these results, bioinformatics analysis provides a potential research direction regarding the interaction between Gal-3 tumor cells and stromal cells, which depends on two potential cell signaling pathways: CCL2-CCR2 and PPIA-BSG.

### Gal-3 induces the transcription of CCL2 and BSG via NFAT1 dephosphorylation

**Figure 3:**
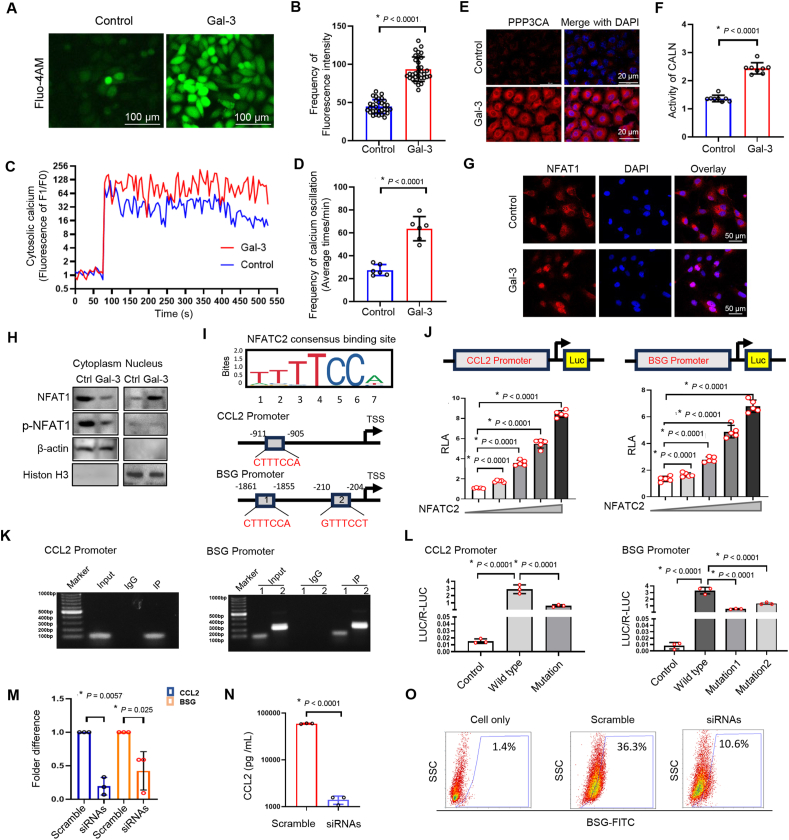
Gal-3 induces the transcription of CCL2 and BSG through NFAT1 dephosphorylation. **(A)** Cytosolic calcium dynamics in HPSCs were monitored via treatment with the calcium dye Fluo-4/AM with or without Gal-3 (50 ng/mL). Gal-3 treatment significantly increased the fluorescence intensity. **(B)** Quantification of calcium intensity in random frame phases revealed the function of Gal-3, shown in Figure 3A. **(C)** Time-lapse imaging of individual cells revealed greater peaks and more frequent calcium oscillations in Gal-3-treated cells than in control cells, indicating a distinct transient calcium phenotype induced by Gal-3. **(D)** Quantification of the number of calcium oscillations per minute in response to Figure 3C. **(E)** The expression of PPP3CA (calcineurin A) was significantly increased in the cytoplasm of Gal-3-treated cells, as shown by imaging flow cytometry. **(F)** CALN activity was similarly increased in Gal-3 genetic HPSCs, confirming enhanced calcium signaling. **(G)** Imaging flow cytometry revealed that Gal-3 treatment induced the translocation of NFAT1 from the cytoplasm to the nucleus. **(I)** JASPAR database enrichment of the potential NFATC2 consensus motif (TTTTCCA) at the promoters of CCL2 and BSG. **(J)** Luciferase assays revealed that NFATC2 activated the transcription of CCL2 and BSG in a dose-dependent manner following co-transfection with the pGL3-CCL2-luc and pGL3-BSG-luc vectors in HEK293T cells. **(K)** Chromatin immunoprecipitation‒PCR results demonstrated that NFATC2 bound to the promoter regions of CCL2 and BSG in HPSCs. Two primers for BSG were designed to separate the two bands at the promoter of NFATC2. **(L)** The dual-luciferase reporter plasmids CCL2 and BSG were co-expressed with NFATC2 in HEK293T cells. **(M)** The efficiency of siRNA-mediated knockdown of NFATC2 in reducing the RNA levels of CCL2 and BSG. **(N)** siNFATC2 significantly reduced CCL2 secretion, as shown by ELISA. **(O)** NFATC2 knockdown decreased the accumulation of BSG on the cell membrane, as determined by a flow cytometry assay. (B), (D), (F), (J), and (L–N) represent the mean ± standard deviation of independent experiments. *p*-values were analyzed via two-tailed unpaired student's *t*-tests for (B), (D), (F), and (N) and one-way ANOVA for (J), (L), and (M). “∗" indicates statistical significance. **Gal-3,** galectin-3; CCL2, C–C motif chemokine 2; PPP3CA, protein phosphatase 3 catalytic subunit alpha; NFATC2, nuclear factor of activated T-cells 2; BSG, basigin; NFAT1, nuclear factor of activated T-cells 1; HPSCs, human pancreatic stellate cells; CALN, calcineurin.

**Fig. S2:**
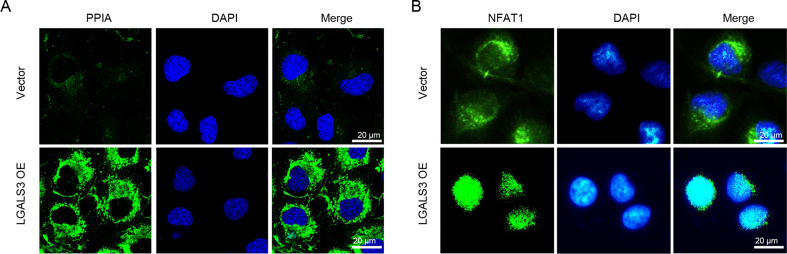


Gal-3, in combination with calcium voltage-gated channel auxiliary subunit gamma 1 (CACNG1), a subunit of L-type voltage-gated calcium channels, affects cytosolic calcium dynamic signals.^19^ In HPSCs treated with the calcium dye Fluo-4/AM with or without Gal-3 (50 ng/mL), a different transient cytosolic calcium phenotype was induced (Fig. 3A). Compared with vehicle, Gal-3 significantly increased the number of calcium spikes in the frame phase images, as indicated by the fluorescence intensity (Fig. 3A and B). Since most parent cells do not strongly react with Fluo-4/AM, we next monitored time-lapse changes in calcium influx in individual cells, which showed high spikes in both groups (Fig. 3C). Interestingly, oscillation of calcium transients was highly common in most Gal-3-treated cells. They appeared more frequently and presented a higher peak value of calcium transients (Fig. 3C, D). [Ca^2+^]i can directly activate Ca^2+^-dependent calcineurin (CALN)^20^ and is dependent on the cyclophilin A–cyclosporin A (CypA/PPIA-CsA) complex.^21^ We subsequently detected the expression of PPP3CA, an important protein for calmodulin-dependent phosphatase activity.^22^ There was significantly high expression of PPP3CA in the rGal-3-treated cells (Fig. 3E). Furthermore, CALN activity presented similar results in Gal-3-producing HPSCs (Fig. 3F). In addition, PPIA was highly expressed in LGALS3-overexpressed HPSCs (Fig. S2A). Many reports indicate that it plays a critical role in the Ca^2+^–calcineurin–NFAT signaling pathway for health and diseases.^23^, ^24^, ^25^ We ascertained the location of NFAT1 transferred from the cytoplasm to the nucleus in HPSCs under rGal-3 treatment (Fig. 3G) and LGALS3-overexpressed genetic cells (Fig. S2B) using immunofluorescence staining. Furthermore, the protein levels of p-NFAT1 were detected in both the cytoplasm and nucleus under conditions of Gal-3 overexpression. The Gal-3-mediated response enhanced the dephosphorylation of NFAT in the nucleus (Fig. 3H). These data confirm that Gal-3 plays an essential driving role in the process from calcium oscillation to CALN activity and then to the transfer of NFAT to the nucleus via dephosphorylation.

NFAT1 might operate via transcription-dependent mechanisms, and multiple transcription factors could be implicated.^26^ Initially, we hypothesized that the consensus binding site of NFATC2, the gene associated with NFAT1, is accessible at promoters spanning from −2000 bp to −10 bp of CCL2 and BSG in the JASPAR database. As presented in Figure 3I, one potential binding region for cis–trans-CCL2 and two potential binding sites for cis–trans-BSG genes had high scores. Next, the pGL3-CCL2-luc vector and the pGL3-BSG-luc vector were co-transfected with pCDNA3.1-Flag-NFATC2 into HEK293T cells at ratios ranging from 1:0.5 to 1:8, and the luciferase activity was subsequently determined. CCL2 and BSG are target genes of NFATC2 in a dose-dependent manner (Fig. 3J). Furthermore, the results of chromatin immunoprecipitation‒PCR revealed that enrichment of NFATC2 occurred at the promoter regions of CCL2 and BSG in HPSCs (Fig. 3K). Additionally, the results of the dual-luciferase assay revealed that NFATC2 increased the luciferase activity of the CCL2-wild-type or BSG-wild-type strains but had little influence on the luciferase activity of the CCL2-mutant or BSG-mutant strains, indicating that NFATC2 activated the transcriptional activity of CCL2 and BSG (Fig. 3L). Furthermore, the RNA levels of CCL2 and BSG transcription were measured using small interfering RNAs targeting NFATC2. Both CCL2 and BSG were inhibited by 60%–80% (Fig. 3M). Ultimately, we noted that siNFATC2-mediated knockdown markedly reduced CCL2 secretion (Fig. 3N) and the accumulation of BSG on the cell membrane (Fig. 3O). These results suggest that a specific motif (TTTTCCA) of NFATC2 within the target protomer is involved in the transcriptional activation of CCL2 and BSG in HPSCs. Overall, we reveal that Gal-3 induces the expression of CCL2 and BSG via calcium-dependent CALN dephosphorylation of FNAT1 and promotes the transcription of CCL2 and BSG in TAFs.

### TAF reduces the oxidative phosphorylation level of tumor cells through the CCL2-CCR2-NOX axis

**Figure 4:**
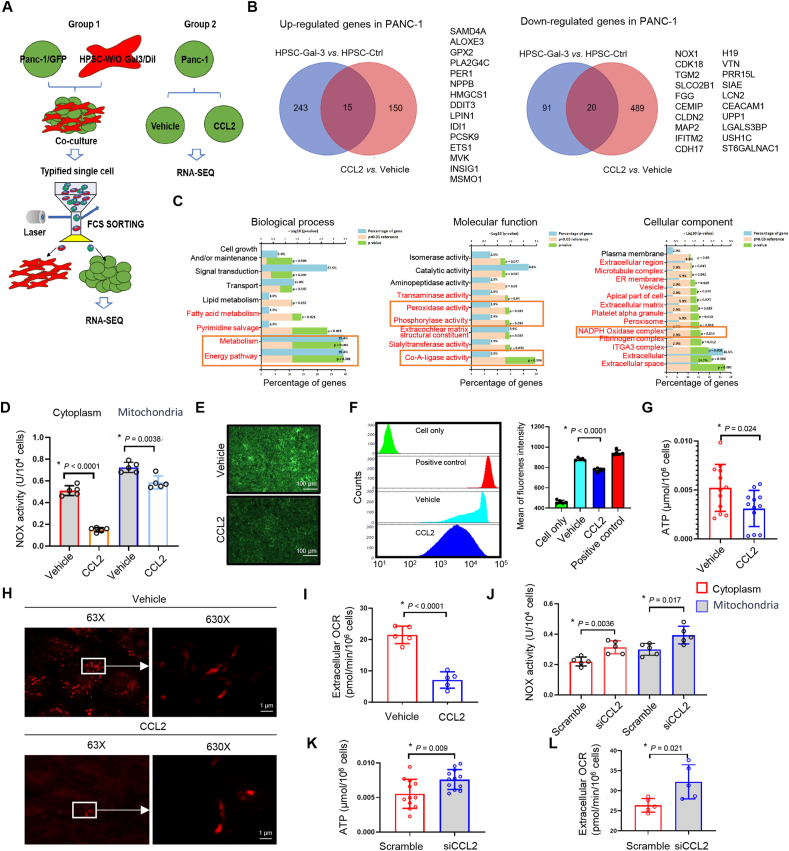
Impact of TAF-mediated signaling on pancreatic adenocarcinoma cells. **(A)** Diagram of the sequencing design. Group 1: Panc-1 cells were co-cultured with pancreatic stellate cells (HPSCs) (with or without galectin-3/Gal-3 treatment for 48 h) and then sorted by flow cytometry to isolate GFP-positive cells. RNA sequencing was performed on the GFP-positive cells. Group 2: RNA sequencing was performed on Panc-1 cells with or without recombinant CCL2 protein treatment. **(B)** A Venn diagram shows 15 co-up-regulated genes and 20 co-down-regulated genes compared with the controls via two groups of sequencing data. **(C)** KEGG pathway analysis of biological process, molecular function, and cellular component panels via FunRich software 3.0. **(D)** An NADPH oxidase activity assay was performed and revealed significant down-regulation of both the plasma membrane and nucleus in Panc-1 cells in response to CCL2. **(E)** Imaging analysis demonstrated that ROS activity was significantly reduced in CCL2-treated Panc-1 cells. **(F)** Flow cytometry detection confirmed a reduction in ROS activity. **(G)** ATP activity was used to detect the amount of ATP produced per 10^6^ cells with or without CCL2 treatment. **(H)** Super-resolution images show the morphology of mitochondria in Panc-1 cells with or without CCL2 via a mitochondrial tracer. **(I)** OCR assay for testing extracochlear O^−^ per 10^6^ Panc-1 cells under CCL2 treatment. **(J)** An NADPH oxidase activity assay was performed and revealed significant down-regulation of both the plasma membrane and nucleus in Panc-1 cells in response to siCCL2. **(K)** ATP activity with or without CCL2 interference. **(L)** OCR assay for Panc-1 under CCL2 interfering treatment. (D), (G), and (I–L) represent the mean ± standard deviation of independent experiments. *p*-values were analyzed via two-tailed unpaired student's *t*-tests. “∗” indicates statistical significance. CCL2, C–C motif chemokine 2; **TAF,** tumor-associated fibroblast; OCR, oxygen consumption rate.

To explore the impact of TAF-mediated PAAD on tumor cells, RNA sequencing was implemented in two manners, with GFP labeling being employed in Panc-1 cells and Dil labeling being utilized in HPSCs. Furthermore, two forms of RNA sequencing were carried out to identify the major and significant genes that affect tumor cells. As shown in Figure 4A, one group consisted of cocultured Panc-1 cells and HPSCs with or without Gal-3 for 48 h, which were characterized as single cells, followed by sorting of the green cells via flow cytometry. Another group involved treating Panc-1 parental cells with or without the recombinant protein CCL2. Compared with those in the controls, 15 genes were co-up-regulated, and 20 genes were co-down-regulated in Panc-1 cells, as shown in Figure 4B. We then analyzed these genes via the Kyoto Encyclopedia of Genes and Genomes (KEGG), metabolism, and energy pathways, and Co-A-ligase, peroxidase, and phosphorylase activities were presented in the molecular function panel. Notably, extracellularly associated complexes and NADPH oxidase complexes were extremely derived in the cellular component (Fig. 4C).

**Fig. S3:**
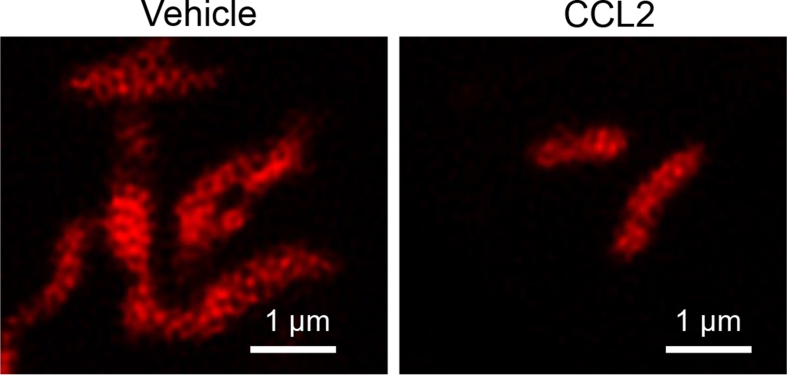


The sequencing data revealed that NADPH oxidase 1 (NOX1) was significantly down-regulated when CCL2 or Gal-3 responded to Panc-1 cells. Although NOX, a ROS-producing enzyme, was initially identified in the membrane of phagocytic cells,^27^ its enzymes are involved in numerous critical physiological processes, including the posttranslational processing of proteins, cellular signaling, and the regulation of gene expression in cancers.^28^ To date, we have identified the intercellular and nuclear NOX activity in CCL2-treated Panc-1 cells. These results suggest that CCL2 might potently block NOX activity both in the cytoplasm and in the mitochondria (Fig. 4D). It is widely acknowledged that NOX induces the aggregation of ROS, adversely affects cell proliferation and cellular mitosis, and promotes apoptosis via extreme superoxide or hydrogen peroxide.^29^ Imaging analyses revealed that ROS activity was directly reduced by CCL2 (Fig. 4E). According to the fluorescence flow cytometry results, the degree of ROS activity decreased by at least one order of magnitude (Fig. 4F). The ATP activity was inhibited by approximately 50%, as was the case for CCL2 (Fig. 4G). Furthermore, a mitochondrial tracer was used to label the intercellular mitochondria, which were then imaged via super-resolution optical laser scanning confocal microscopy (LSM980, Airyscan2, ZEISS). CCL2 reduced the number of mitochondria and increased mitochondrial disaggregation (Fig. 4H; Fig. S3). The oxygen consumption rate assay was further performed, as shown in Figure 4I, and CCL2 inhibited the oxygen consumption of the tumor cells. To further investigate the role of CCL2 in HPSCs, interfering RNA was employed to knock down CCL2 expression. As a result, NOX activity was elevated in both the mitochondria and cytoplasm (Fig. 4J), while ATP production increased significantly in the siRNA CCL2 group compared with the control group (Fig. 4K). Moreover, oxygen consumption rate levels in the siRNA CCL2 group were approximately doubled relative to those in the control group (Fig. 4L). These data demonstrate that the reduction in the low oxidative phosphorylation level resulting from NOX inhibition is attributed to the Gal-3-induced CCL2 secretion provided by TAFs.

### Gal-3 enhances drug resistance to gemcitabine

**Figure 5:**
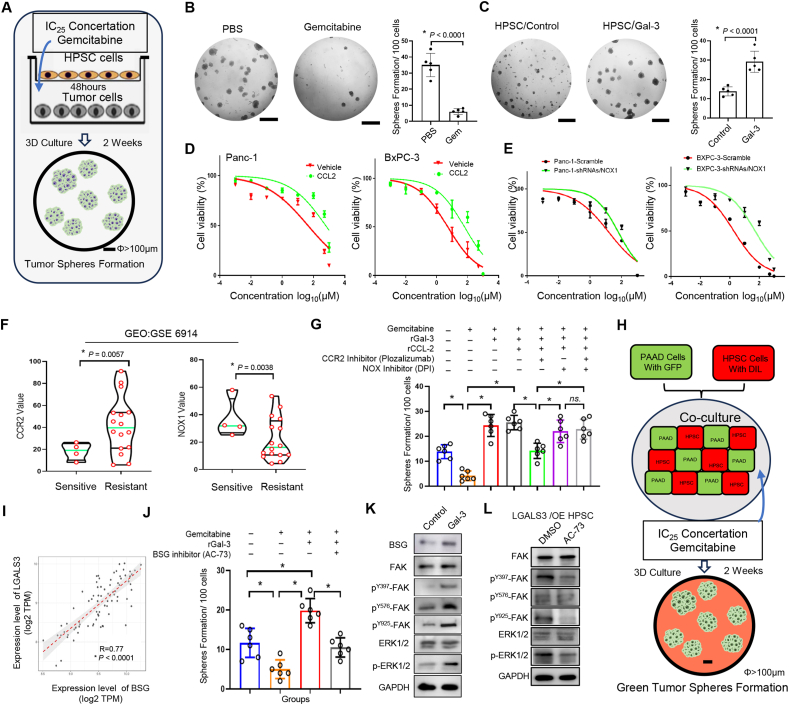
Gal-3 enhances drug resistance to gemcitabine in pancreatic cancer. **(A)** A comprehensive coculture model of tumor cells and TAFs was developed to assess the impact of gemcitabine treatment on tumor formation via low-attachment 96-well plates and Boyden chambers. **(B)** Tumor sphere formation after treatment with 10 μmol/L gemcitabine. **(C)** Tumor sphere formation by co-culturing Panc-1 cells and HPSCs with or without Gal-3 and treated with 10 μmol/L gemcitabine. **(D)** Gemcitabine cytotoxicity experiments in Panc-1 (unsensitive) and BxPC-3 (sensitive) cells revealed that CCL2 treatment increased the gemcitabine IC50 in Panc-1 cells. **(E)** NOX1 knockdown in both Panc-1 and BxPC-3 cells affected by gemcitabine resistance. **(F)** Bioinformatics analysis of data from the GEO database (GES: 6914) confirmed that CCL2 and NOX1 contribute to gemcitabine resistance. **(G)** Tumor sphere formation ability of a co-culture system containing Gal-3, CCL2, a CCR2 inhibitor (plozalizumab), and a NOX1 inhibitor (diphenyleneiodonium chloride). **(H)** Model for co-culture with distinct fluorescent labels. **(I)** A positive correlation between BSG and Gal-3 expression was detected via TCGA database analyses. **(J)** Tumor sphere formation in response to the administration of AC-73 (a BSG-specific inhibitor) upon rGal-3 stimulation. **(K)** Western blotting was used to analyze the protein level of BSG and the phosphorylation response to the FAK-ERK pathway. (B, C, G, J) represent the mean ± standard deviation of independent experiments. *p*-values were analyzed via two-tailed unpaired student's *t*-tests for (B) and (C) and one-way ANOVA for (G) and (J). “∗" indicates statistical significance. Gal-3, galectin-3; CCL2, C–C motif chemokine 2; CCR2, C–C motif chemokine receptor 2; TAFs, tumor-associated fibroblasts; HPSCs, human pancreatic stellate cells; NOX1, NADPH oxidase 1; BSG, basigin; FAK, focal adhesion kinase; ERK, extracellular signal-regulated kinase.

Because gemcitabine is a nucleoside metabolic inhibitor used as a major chemotherapy agent in the treatment of pancreatic cancer,^30^ we generated a comprehensive and elaborate model for accurately evaluating tumor formation after gemcitabine treatment through the co-culture of tumor cells with TAFs (Fig. 5A). Using low-attachment 96-well plates and a Boyden chamber, we verified the effectiveness of this system and finally determined the optimal concentration of gemcitabine for use. Treatment with 10 μM gemcitabine for 2 weeks significantly inhibited tumor sphere formation, with an approximately 80% inhibition rate (Fig. 5B). When Panc-1 cells and HPSCs were co-cultured with or without Gal-3, we discovered that HPSCs could increase the number of tumor spheres by twofold in comparison with the situation in which Panc-1 cells were treated with gemcitabine alone, and that the number of HPSCs genetically expressing Gal-3 could further double the number of tumor spheres in contrast to HPSCs alone (Fig. 5C).

For the gemcitabine cytotoxicity experiments, we found that the expression of LGALS3 was highly correlated with the concentration of gemcitabine IC50 value from analyzing the Genomics of Drug Sensitivity in Cancer (GDSC) database^31^ (Fig. S3). Panc-1 cells, which are relatively highly resistant, and BxPC-3 cells, which are relatively sensitive,^32^ were used to test the gemcitabine IC50 value after rCCL2 treatment. CCL2 increased the IC50 value of Panc-1 cells from 44.1 ± 1.28 μM to 600.3 ± 3.15 μM. Compared with vehicle-treated cells, BxPC-3 cells presented an increase in the IC50 value from 5.14 ± 0.48 μM to 60.34 ± 2.12 μM (Fig. 5D). Similar to the response of rCCL2 to gemcitabine-induced cytotoxicity, NOX1 knockdown in both Panc-1 and BxPC-3 cells via shRNAs resulted in identical outcomes in terms of gemcitabine drug resistance (Fig. 5E). These data can be validated by analyzing bioinformatics datasets from GEO (GES: 6914). CCL2 is associated with significantly greater resistance to gemcitabine. In contrast, NOX1 cells displayed notably increased sensitivity to gemcitabine (Fig. 5F). The efficacy of gemcitabine and the response to Gal-3 in Panc-1 and BxPC-3 cells were detected in our previous report. As depicted in Figure 5G, Gal-3 plus CCL2 and the combination of a CCR inhibitor (neutralizing antibody plozalizumab) and/or a NOX1 inhibitor (diphenyleneiodonium chloride) were used to test gemcitabine drug resistance via the co-culture system. Both Gal-3 and CCL2 enhanced the formation of tumor spheres, and this process was inhibited by the CCR2 inhibitor. However, the ability of the NOX inhibitor to promote tumor sphere formation was restored. Notably, the inhibition of NOX is not completely contingent upon the Gal-3-CCL2-CCR2 pathway. It lies at the substrate end of the molecular chain of the indirect tumor–stromal interaction via the secretion of proinflammatory mediators within the microenvironment.

Considering the direct inference through the PPIA-BSG interaction between the membrane of the tumor and stromal cells,^33^ a novel model for co-culture with distinct fluorescent labels has been established (Fig. 5H). An analysis of the positive correlation between BSG and Gal-3 from the TCGA database (Fig. 5I) revealed that the administration of AC-73 (a specific inhibitor of BSG) and gemcitabine markedly diminished the formation of tumor spheres upon rGal-3 stimulation (Fig. 5J). The results of the western blotting analysis indicated that Gal-3 activated BSG in response to the phosphorylation of the focal adhesion kinase (FAK)-extracellular signal-regulated kinase (ERK) pathway (Fig. 5K). In addition, treatment of LGALS3-overexpressing HPSCs with the BSG inhibitor AC-73 resulted in a significant reduction in the phosphorylation levels of FAK and ERK (Fig. 5L). These findings suggest that Gal-3 enhances drug resistance to gemcitabine in two ways. One relies on the influence of CCL2-CCR2 signaling via inflammatory factor secretion in the tumor microenvironment, and the other is dependent on the interaction of membrane receptors with the BSG-FAK-ERK pathway.

### Gal-3 inhibition combined with gemcitabine reduces pancreatic cancer cell growth

**Figure 6:**
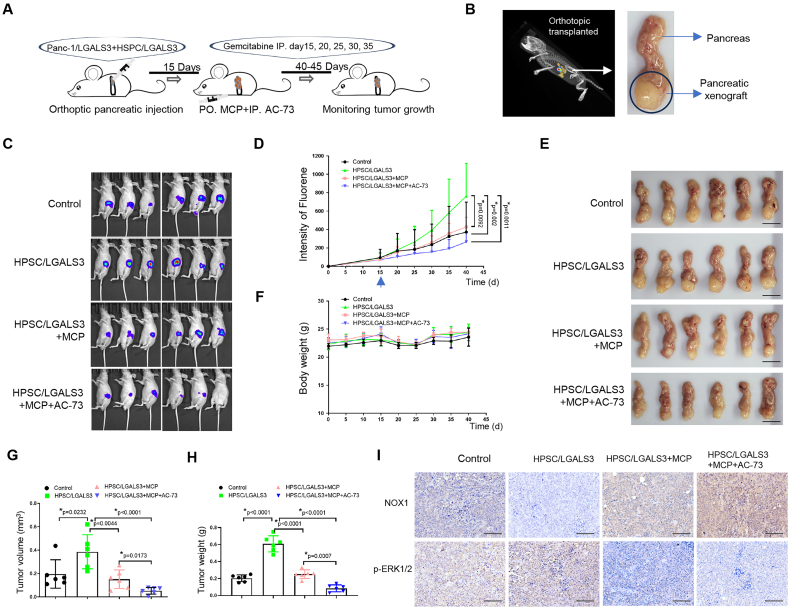
*In vivo* experiments confirmed that inhibiting Gal-3 combined with gemcitabine reduces pancreatic cancer cell growth. (A) Schematic description of the drug combination experimental design in mice. **(B)** Images of the orthotopic xenograft model by 3D detection and tumor growth in the tail of the pancreas. **(C)** Mice with luciferase-labeled Panc-1 cells and HPSCs were treated with gemcitabine, and tumor growth was monitored via bioluminescence imaging. *n* = 6. **(D)** Quantification of the fluorescence intensity of the mice in each group. The blue arrow indicates the start day of drug treatment. **(E)** At the end of the *in vivo* xenograft study, the whole pancreas, including the transplanted tumors, was removed for analysis. Bar = 1 cm. **(F)** Body weights of mice were measured every 5 days. **(G, H)** Quantification of the tumor volume (G) and weight (H) in different groups of mice. **(I)** Immunohistochemical images show NOX1 and p-ERK expression in tumors from different groups. Bar = 200 μm “∗” indicates statistical significance. Gal-3, galectin-3; NOX1, NADPH oxidase 1; HPSCs, human pancreatic stellate cells; p-ERK, phosphorylated extracellular signal-regulated kinase.

Using an orthotopically injected immunodeficient mouse xenograft model, we assessed the potency of the combination of gemcitabine and a Gal-3 inhibitor (MCP) and/or BSG/CD147 inhibitor (AC-73) *in vivo*. The experimental procedure is shown in Figure 6A. Luciferase-labeled Panc-1/LGALS3 OE cells with or without HPSC/LGALS3 OE cells were orthoptic injected into the pancreatic tail of mice via a G33-needle (Hamilton S600, Switzerland). The mice were harvested with MCP (orally, 400 mg/kg/day); AC-73 (20 mg/kg) was administrated intraperitoneally three times a week; gemcitabine (50 mg/kg) injection was given intraperitoneally to each mouse every 5 days (a maximum of 5 times) after 14 days of implantation. Tumor progression was monitored every 5 days via a noninvasive real-time cooled bioluminescence imaging system. The whole pancreas, including the transplanted tumors, was removed at the end of the *in vivo* xenograft (Fig. 6B). Continuous monitoring of tumor growth revealed that HPSC/LGALS3 overexpression accelerated pancreatic tumor growth, counteracting the effects of gemcitabine therapy. In contrast, MCP administered alone and in combination with AC-73 were effective in slowing tumor growth in both tumor and stromal growth environments (Fig. 6C). When AC-73 was combined with MCP and gemcitabine, significant inhibition of tumor growth was observed (Fig. 6D). The combination therapy with gemcitabine further significantly suppressed the progression of pancreatic cancer (Fig. 6E) without any toxicity-related adverse effects, such as weight loss (Fig. 6F). Quantitative evolution of tumor burden indicated that down-regulation of Gal-3 formed much smaller tumors than the control group, which was in accordance with the volume and wet weight of the tumors, as measured and shown in Figure 6G and H. Immunohistochemistry for NOX1, and p-ERK expression was performed in tumors from each group. The expression of NOX1 was significantly prominent in the cytoplasm of tumors in the MCP plus AC-73 group, while p-ERK expression was reduced in the same one (Fig. 6I). These results suggest that Gal-3 inhibition *in vivo* can effectively potentiate the anti-tumor effect of gemcitabine.

## Discussion

In previous studies, we have demonstrated that Gal-3 is overexpressed in pancreatic cancer, while the secreted form of Gal-3 plays a crucial role in regulating the secretion of inflammatory factors by stromal cells, namely, TAFs, through the cell membrane receptors of integrin family members.^12^ Because the secretion of inflammatory factors may regulate cancer-driving genes and promote the progression of pancreatic cancer,^34^ blocking these pathways can effectively control the growth of tumor cells. However, pancreatic cancer is a clinically refractory tumor with limited pharmacotherapy options. Gemcitabine, as a first-line treatment, is a key therapeutic choice. Therefore, our research aimed to investigate the synergistic action of Gal-3 in the gemcitabine therapy of pancreatic cancer from the perspective of the tumor microenvironment. Increased Gal-3 can increase the IC50 value of gemcitabine both *in vivo* and *in vitro*, suggesting that Gal-3 could also be a target for combination therapy in pancreatic cancer.

Numerous reports have shown that Gal-3 serves as a potential biomarker for risk stratification in patients with pancreatic cancer, identifying individuals at increased risk of this disease.^35^ It can guide personalized management strategies for pancreatic cancer, including enhanced monitoring or early intervention strategies.^36^ Therefore, intervention with Gal-3 expression has positive effects on pancreatic cancer treatment. Moreover, our interest lies in the association of Gal-3 expression with TAFs. At the beginning of this study, through immunohistochemical and bioinformatics analyses, we discovered that LGALS3^+^ tumor cells were highly correlated with fibroblasts via the PPIA-BSG pathway. Next, active TAFs are fed back to tumor cells through the secretion of CCL2 and its receptor CCR2 on the membrane of LGALS3^+^ cells. Subsequently, PPIA/CypA is a specific cytosolic binding protein that induces chemotaxis independent of the ectodomain of BSG.^37^^,^^38^ Indeed, numerous reports have demonstrated that PPIA is up-regulated in most cancers, as shown in Figure 2E, and serves as a key determinant of malignant transformation and metastasis.^38^^,^^39^ Finally, to date, we have identified inflammatory factors produced by stromal cells, such as CCL2.

To explore the mechanism involved in this process, an important role of recruiting Ca^2+^-dependent interactors and regulators was considered. First, the influx and oscillation of intercellular calcium were initially assessed in HPSCs by Gal-3 induction. The mechanism of the Ca^2+^–calcineurin–NFAT signaling pathway was apparently in accordance with the Gal-3 stimulation of TAFs and co-culture with PAAD cells. Second, we identified that Ca^2+^-sensitive calcineurin can dephosphorylate NFAT1, which can accumulate in the nucleus and promote transcriptional activity.^25^ Many reports have illustrated the critical role of Ca^2+^-dependent proteins such as apoptosis-linked gene-2 (ALG-2), which are recruited to damaged lysosomes^40^; protein kinase C alpha (PKCα), which regulates Golgi fragmentation and increases intra-Golgi transport^41^; and secretory pathway calcium ATPase 1 (SPCA1), a primarily trans-Golgi-localized Ca^2+^ ATPase.^42^ Calcium ions directly give rise to physiological alterations in cellular organelles. Third, we have not yet directly validated the changes in cellular organelles within the TAF. Instead, a potential pathway for oxidative phosphorylation within tumor cells through tumor–stromal interactions, especially the CCL2-CCR2 signaling pathway, was identified. In this study, we found that both Gal-3 and CCL2 led to an enrichment of oxidative phosphorylation-associated genes. Moreover, CCL2-CCR2 signaling could reduce NOX activity. NOX is a complex multidomain protein oxidase that catalyzes NADPH to produce one-electron transmembrane transfer to molecular oxygen and activate ROS, which is dependent on mitochondrial oxidative phosphorylation.^28^^,^^43^ Here, we investigated NOX activity, ROS levels, and mitochondrial ATP production via rCCL2 treatment. According to our report, CCL2 facilitates gemcitabine resistance; conversely, NOX activity increases the sensitivity of cells to gemcitabine chemotherapy. Ana P. Kutschat and colleagues reported that transcriptomic events associated with gemcitabine resistance led to an impaired endoplasmic reticulum stress response, which was dependent on calcium influx in the T cells of PAAD.^44^ Our findings indicate that the calcium influx-induced secretion of inflammatory factors from TAFs is another significant factor contributing to gemcitabine drug resistance.

**Figure 7:**
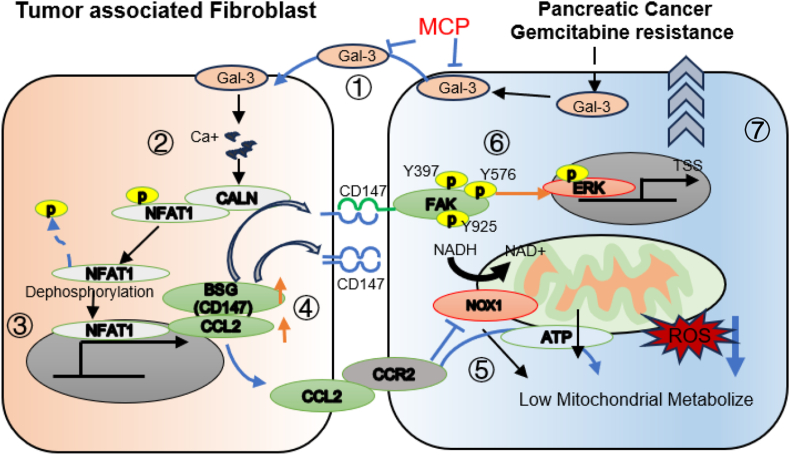
The schematic depicts a proposed model for the function of Gal-3 in response to tumor–stromal interactions in pancreatic adenocarcinoma. Gal-3, galectin-3; CALN, calcineurin; NOX1, NADPH oxidase 1; BSG, basigin; CCL2, C–C motif chemokine 2; CCR2, C–C motif chemokine receptor 2; FAK, focal adhesion kinase; ERK, extracellular signal-regulated kinase; NFAT1, nuclear factor of activated T-cells 1; MCP, citrus pectin extract.

We could draw the diagram of this study. A robust connection was observed between PAAD tumor cells highly expressing Gal-3 and TAFs. On the one hand, within the TAF, calcium ions orchestrate the activity of calcineurin to affect the dephosphorylation of NFAT; NFAT protein subsequently enters into the nucleus for transcription, thereby governing CCL2 and BSG (CD147) signaling. On the other hand, Gal-3-positive tumor cells receive the CCL2-CCR2 signal, which leads to a decrease in the catalytic activity of NOX, culminating in decreases in the ROS and OXPHS levels. Furthermore, Gal-3 can increase the expression of CD147 in both tumor and fibroblasts and activate the FAK-ERK pathway. The ERK pathway further enhances the resistance to gemcitabine.^45^ These up- and down–regulatory processes culminate in resistance to gemcitabine (Fig. 7).

Controlling mitochondrial metabolism is a therapeutic approach to overcoming gemcitabine resistance in PAAD.^46^ In this study, the natural Gal-3 inhibitor-modified MCP effectively regulated the progression of pancreatic cancer, which might be associated with the mechanism of gemcitabine action or regulatory signaling pathways. Based on the metabolic reprogramming of oxidative phosphorylation mechanisms, we confirmed that inhibiting Gal-3 could effectively diminish the dependence of pancreatic cancer on gemcitabine, effectively counteract tumor growth, and reduce related adverse drug reactions. Compared with intravenous injection, oral MCP represents a simpler route of administration. In our experiments, MCP was confirmed to prohibit tumor growth *in vivo*, but it may not directly kill tumor cells *in vitro*. These phenomena may be explained by a competitive effect, in which MCP occupies the cell membrane receptors that bind to Gal-3, rendering the Gal-3-induced cellular signaling pathway ineffective. In our previous reports, MCP inhibited downstream signaling pathways of integrin β1 (ITGB1), reducing the effectiveness of tumor-driving genes. Additionally, reports have shown that MCP can reduce renal and cardiac toxicity associated with drug reactions.^36^^,^^47^ Therefore, MCP is an effective competitive inhibitor of Gal-3 and deserves further investigation. As we know, the natural production and extraction of MCP, along with its associated patents, such as no. 8916541 with the U.S. patent and ZL 201110200381.4 with the Chinese patent, have been prominently displayed in the health supplement markets of both the United States and China for many years. We have investigated the potential role of MCP (provided by Centrax International Corporation, San Francisco, USA) in tumor treatment, thereby providing valuable insights for expanding its applications. Due to the characteristic of MCP as a pure natural preparation, when combined with gemcitabine or targeted inhibitors for treatment, it may reduce the risk of their cytotoxic effects.^12^ Based on these reports, we believe that the potential of MCP compounds to enhance the efficacy and mitigate the side effects of conventional anti-cancer therapies, such as chemotherapeutic agents' activation (*e.g.*, paclitaxel^48^ and gemcitabine^12^), targeted growth factor inhibitors (*e.g.*, STAT3^48^ and BSG), and radiation therapy,^49^ is highly significant. Besides MCP, galectin-3 antagonist 1^50^ and TD139,^12^ which act as galectin-3 inhibitors, also demonstrate significant anti-cancer strategies. Although we did not perform animal experiments with AC-73 as a single agent, this omission does not compromise the inhibitory effect of the combination of MCP and AC-73.

In this study, we generated an orthotopic xenograft mouse model of pancreatic cancer, which allowed for a better understanding of the tumor microenvironment and drug efficacy *in situ* for pancreatic tumor growth. This tumor-*in-situ* xenograft mouse model also provides the most human-like observations for drug delivery, drug metabolism, and adverse drug reactions, which represents the closest organ-specific simulation of tumor growth in animal experiments for pancreatic cancer.

In summary, Gal-3 may serve as a therapeutic target in pancreatic cancer. Gal-3 can increase the IC50 value of gemcitabine by attenuating the sensitivity of Panc-1 and BxPC-3 cells to chemotherapy. We found that Gal-3 expression was regulated not only in tumor cells but also in stromal cells. In the tumor microenvironment, inhibiting Gal-3 in combination with gemcitabine represents a valuable innovation in the pharmacological treatment of pancreatic cancer. Furthermore, owing to the homology of medicine and food, as a natural food-based “drug”, MCP holds potential research and development value.

## Conclusion

Gemcitabine drug-resistant Gal-3-high tumor cells can increase the expression of CCL2 and BSG in TAFs through the Ca^2+^–calcineurin–NFAT pathway, thereby leading to mitochondrial oxidative phosphorylation suspension via CCL2–CCR2 cell signaling. Then, CCR2 activates the FAK-ERK phosphorylation pathway to enhance the resistance to gemcitabine. Blocking the tumor–stromal interaction with MCP could improve the efficacy of PAAD chemotherapy.

## CRediT authorship contribution statement

**Yaheng Wu:** Writing – original draft, Project administration, Investigation. **Guo An:** Methodology, Investigation, Data curation. **Jia Tong:** Writing – original draft, Methodology, Formal analysis, Data curation. **Wenlong Zhang:** Methodology. **Zhihua Tian:** Methodology. **Bin Dong:** Methodology. **Xijuan Liu:** Methodology. **Lin Zhao:** Methodology. **Chunxiang Ye:** Resources, Methodology. **Jingtao Liu:** Writing – original draft, Validation, Supervision, Project administration, Investigation, Funding acquisition, Data curation, Conceptualization. **Wei Zhao:** Writing – review & editing, Writing – original draft, Visualization, Validation, Supervision, Project administration, Investigation, Formal analysis, Data curation, Conceptualization. **Huachong Ma:** Writing – review & editing, Supervision, Project administration, Funding acquisition, Conceptualization.

## Ethics declaration

All the mouse experiments were conducted at Peking University Cancer Hospital (license number: SYXK 2016-0015), conformed to the regulatory standards of Peking University Cancer Hospital regarding Laboratory Animal Care, were approved by the Ethics Committee (EAEC2018-14), and were performed in accordance with the National Institutes of Health Guide (Guide for the Care and Use of Laboratory Animals, 2011). This study was conducted in accordance with the principles of the Declaration of Helsinki. The mice were rapidly sacrificed by euthanasia with CO_2_ without suffering. The acquisition and use of patient tissues were permitted based on the acquisition of informed consent according to the protocol approved by the Ethics Committee of the Beijing Chaoyang Hospital of Capital Medical University (no. 2018-k-99).

## Data availability

All original data are archived and stored at Peking University Cancer Hospital, Beijing, China. The cell lines generated in this study and the controls, including the Panc-1 and HPSC cell lines, will be made available to other researchers upon request.

## Funding

This study was funded by the Noncommunicable Chronic Diseases-National Science and Technology Major Project (China) (No. 2024ZD0520200), the 10.13039/501100001809National Natural Science Foundation of China (No. 81802353), the Foundation of China Association for Promotion of Health Science and Technology (No. JKHY2024003), the Science Foundation of Peking University Cancer Hospital (China) (No. A003004), and the Research Enhancement Fund of Shandong Provincial Hospital (China) (No. 2024TS12).

## Conflict of interests

The authors declare that they have no known competing financial interests or personal relationships that could have appeared to influence the work reported in this paper.

## Figures and Tables

**Fig. S4:**