# Supplementary material for: Galectin-3 in tumor-stromal cells enhances gemcitabine resistance in pancreatic adenocarcinoma by suppressing oxidative phosphorylation
Source: Genes Dis. 2025 May 29;12(5):101702. doi: 10.1016/j.gendis.2025.101702 (PMC12211844; doi:10.1016/j.gendis.2025.101702)
Supplement: Multimedia component 1 [file mmc1.docx]

**Supplemental Methods:**

**Bioinformatics analysis**

The RNA expression profiles of PAAD (n=178) including 146 PDAC samples and 32 non-PDAC PAAD samples were obtained from the TCGA database, while the corresponding clinical data were sourced from the TCGA database and summarized by LinkedOmics publicly available portal analysis (http://www.linkedomics.org/). 211 PAAD protein samples including 137 tumor samples and 74 normal samples from the Clinical Proteomic Tumor Analysis Consortium (CPTAC), were analyzed by The University of Alabama at Birmingham CANcer data analysis Portal (https://ualcan.path.uab.edu/). Additionally, we utilized scRNA-seq data to identify the key genes influencing Gal-3 expression in various cells in tumors, which acquired an external independent validation cohort by downloading the single-cell dataset: GES:111627; 141017; 148673; 154763; 154778; 158356; 162708; 165399 from the GEO database. The canonical correlation analysis (CCA) algorithm was applied to integrate single-cell transcriptomic data from different samples.

**Multiplex immunohistochemistry (mIF) and hematoxylin and eosin (H&E) staining**

Primary PAAD tissues were provided from patients who underwent duodenopancreatectomy or pancreato-splenectomy in the Department of General Surgery, Beijing ChaoYang Hospital. The acquisition and use of these tissues were permitted based on the acquisition of informed consent according to the protocol approved by the Ethics Committee of Beijing ChaoYang Hospital (no. 2018-K-99). 5-μm formation-fixed paraffin-embedded slides were prepared for multiplex immunohistochemistry staining. Briefly, sections were deparaffinized and subjected to heat-induced epitope retrieval in Tris buffer (pH 8.0). Next, a 4-plex panel (Gal-3, SMA, PanCK, and DAPI) was designed. Anti-rabbit/mouse TSA HRP (Panovue, Beijing) was used as the secondary antibody. Each biomarker was visualized and performed using tyramine signal amplification-conjugated fluorophores (PerkinElmer, USA). The number of non-overlapping regions of interest per slide. For hematoxylin and eosin staining, tissues were fixed, and staining was performed based on the protocol described previously^1^.

**Preparation of Modified Citrus Pectin (MCP) and recombinant proteins and inhibitors**

As a natural inhibitor of Gal-3, MCP was modified to specifically inhibit the binding of Gal-3 to the cell surface and competitively inhibit Gal-3-activated signaling pathways, as described previously^2^. rGal-3 (product: #450-38-1MG) was purchased from PeproTech company; rCCL2/MCP-1 (HY-P78173), CCR2 inhibitor Plozalizumab (MLN-1202, 1610761-46-0), NOX inhibitor Diphenyleneiodonium chloride (DPI, 4673-26-1), BSG inhibitor AC-73 (775294-71-8) were purchased from MedChemExpress company; the plasmid of siNFATC2 (sc-36055) and shRNA NOX1 (sc-43939-SH) were purchased from Santa Cruz Biotechnology.

**Cell Proliferation Assay**

Cell viability and proliferation were assessed using the "Cell^Titer^ Aqueous One Solution Cell Proliferation Assay" kit from Promega, following the manufacturer's instructions. Absorbance at 490 nm was measured using a multimode microplate reader (BMG Labtech ClarioStar),

**The half-maximal inhibitory concentration (IC50) assay**

IC50 was depicted through a concentration-response curve, with the concentration of the compound serving as the horizontal coordinate and the percentage of cell viability as the vertical coordinate. In this concentration-response curve, the biological process or activity was inhibited by 50% when the concentration reached the IC50, and inhibitory concentration 50% (IC50) values were calculated using nonlinear regression analysis of GraphPad Prism software (version 7.0).

**Calcineurin, OCR, ATP, ROS, and NOX activity assays**

According to the manufacturer's instructions, a CALN Activity Assay Kit (SEK-030024486, G-clone, Beijing, China), an Oxygen Consumption Rate (OCR) Assay Kit (E297, Dojindo Molecular Technologies, Inc.) an ATP Content Assay Kit (BC0300), a NOX Activity Assay Kit (BC0630) and Reactive Oxygen Species Assay Kit (CA1410) from Solarbio (Beijing, China) were used to measure variations in intracellular calcineurin activity, extracellular OCR based on the Stern-Volmer equation., ATP production, NOX activity both intracellular plasma and nuclear and ROS release activity between vehicle and regents.

**Cytokine Antibody Array Assay**

rGal-3-treated or untreated HPSC were collected by serum-free conditioned medium, and cytokine antibody arrays were employed for testing the secretion of cytokines and chemokines, following the manufacturer's instructions (human cytokine array 3, MA6150; Panomics, Redwood City, CA,)^3^.

**Immunoblot analysis**

Cells were harvested and lysed in a RIPA lysis buffer (consisting of 50 mM Tris HCl, 150 mM NaCl, 1% NP-40, 0.5% Sodium Deoxycholate, 1.0 mM EDTA, 0.1% SDS, and 0.01% sodium azide, pH 7.4). After mixing with a sample loading buffer containing 2-mercaptoethanol (2ME) and boiling. The same concentration proteins of the samples were loaded and separated by SDS-PAGE. A 0.45μM PVDF membrane (Merck Millipore, Hertfordshire, UK) was used to be transferred for protein further analysis. The membrane was incubated with the right primary antibodies: BSG (Abcam, #ab108308), GAPDH (CST, #2118), FAK (CST, #13009), p-Y397-FAK (CST, #8556), p-Y576-577-FAK (CST, #3281), p-Y925-FAK (CST, #3284), ERK1/2 (Santa Cruz Biotechnology, # sc292838), p-ERK1/2 (Santa Cruz Biotechnology, # sc7383), respectively, washed, and incubated with the secondary antibody conjugated with HRP. The proteins were visualized ECL detection reagents (Millipore, Billerica, MA, USA) and captured by AI600 version 1.2.0 on an Amersham Imager 600 (GE Healthcare, Chicago, IL, USA).

**Immunohistochemistry**

Slides with xenograft tissues from Gal-3 genetic Panc-1+HPSCs were processed through de-paraffinization, rehydration, and peroxidase quenching. Antigen retrieval was done using citric acid buffer and microwaving. Respectively blocking and primary antibody (Cleaved casepase-3, CST, #9661; p-ERK1/2, Santa Cruz Biotechnology, # sc7383; NOX1, Origene, SKU AP08314PU-N) incubation were followed by Poly-HRP anti-rabbit IgG antibody incubation. DAB staining and hematoxylin counterstaining were performed, and the slides were prepared for microscopic examination as descripted in our pervious report^4^.

**The luciferase reporter assay**

HEK 293T cells were seeded in a 24-well plate and subjected to co-transfection with targets wild type and mutation sequences reporter plasmids alongside a normal control plasmid. The binding sites (predicted by JASPAR) were amplified by PCR and inserted immediately downstream of the firefly luciferase cDNA in the pGL3-control vector (Promega, Madison, WI, USA) to construct pGL3-CCL2 or pGL3-BSG wide type and mutations. Briefly, 10^5^ HEK 293T cells per well were seeded in 24-well plates, and 300 ng of pGL3 constructs plus 26 ng of pRL-TK plasmid that expressed Renilla luciferase were co-transfected with 1μg of NFATC2 using 3μg PEI. After transfection for 48 hours, the luciferase activity was measured using a Dual-Luciferase Assay kit (Promega). The data for each sample were normalized to Renilla luciferase activity, and three independent experiments were performed^5^.

**Indirect immunofluorescence staining**

HPSC and genetic cells were cultivated for 48 hours with diverse doses of Gal-3. Subsequently, after being fixed with 4% paraformaldehyde and permeabilized using 0.5% Triton X-100, the subjects underwent indirect immunofluorescence staining with PPP3CA (1:100) (EPR24997-22, Abcam) and NFAT1 (1:100) and PPIA (1:100) (ab58144, Abcam) primary antibodies, followed by secondary antibodies labeled with Alexa-488 or Rhodamine in the darkness as described elsewhere. The nuclei were stained with 4,6-diamidino-2-phenylindole dihydrochloride (DAPI, Polysciences, Warrington, PA, USA) at 0.5 μg/mL for 5 minutes. All specimens were mounted in 90% glycerol/PBS containing 2.5% 1,4-diazabicyclo (2, 2, 2) octane and imaged by means of laser scanning confocal microscopy ((LSM980, Airysan2, ZEISS, Wetzlar, Germany).

**Refences**

1. Sun Y, Dong Y, Liu X, et al. Blockade of STAT3/IL-4 overcomes EGFR T790M-cis-L792F-induced resistance to osimertinib via suppressing M2 macrophages polarization. EBioMedicine 2022;83:104200.

2. Nangia-Makker P, Hogan V, Honjo Y, et al. Inhibition of human cancer cell growth and metastasis in nude mice by oral intake of modified citrus pectin. J Natl Cancer Inst 2002;94:1854-62.

3. Zhao W, Ajani JA, Sushovan G, et al. Galectin-3 Mediates Tumor Cell-Stroma Interactions by Activating Pancreatic Stellate Cells to Produce Cytokines via Integrin Signaling. Gastroenterology 2018;154:1524-1537.e6.

4. Ma S, Xie F, Wen X, et al. GSTA1/CTNNB1 axis facilitates sorafenib resistance via suppressing ferroptosis in hepatocellular carcinoma. Pharmacol Res 2024;210:107490.

5. Ma Y, Yang X, Zhao W, et al. Calcium channel α2δ1 subunit is a functional marker and therapeutic target for tumor-initiating cells in non-small cell lung cancer. Cell Death Dis 2021;12:257.

**Figure S1** Enlarged images of galectin-3 (Gal-3) expression, in response to Figure 1G.

**Figure S2** PPIA and NFAT1 expression and location in HPSCs by immunofluorescence. **(A)** High PPIA expression in the cytoplasm of LGALS3 OE HPSCs. **(B)** Imaging flow cytometry was performed using an NFAT1 antibody in HPSCs with genetic overexpression of LGALS3. Bar = 20 μm. LGALS3, lectin galactoside-binding soluble 3; PPIA, peptidylprolyl isomerase A; NFAT1, nuclear factor of activated T-cells 1; HPSCs, human pancreatic stellate cells.

**Figure S3** Images of the super-resolution morphology of mitochondria obtained via a mitochondrial tracer. In response to Figure 4H, the images are magnified 1000 times following the treatment of Panc-1 cells with rCCL2. Bar = 1 μm. rCCL2, recombinant C-C motif chemokine 2.

**Figure S4** The correlation between LGALS3 expression and gemcitabine IC50 value in pancreatic cancer cells. Using GDSC data from Wellcome Sanger Institute, Massachusetts General Hospital Cancer Center (USA), twenty-six pancreatic cancer cell lines (AsPC-1, BxPC-3, CAPAN-1, CAPAN-2, CFPAC-1, HPAC, HPAF-II, HuP-T3, HuP-T4, KP-1N, KP-2, KP-4, MIA-PaCa-2, PA-TU-8902, PA-TU-8988T, PANC-02-03, PANC-03-27, PANC-04-03, PANC-08-13, PANC-10-05, PL4, PSN1, SU8686, SUIT-2, SW1990, YAPC) were analyzed for their IC50 responses to gemcitabine. A cutoff of 50% was used, and the Mann-Whitney U test was employed for the evaluation of significant differences. "*" indicates statistical significance. LGALS3, lectin galactoside-binding soluble 3.
